# Nutritional risk and cancer pain as determinants of radiotherapy-induced severe lymphocytopenia: development and validation of a nutrition-integrated predictive nomogram

**DOI:** 10.3389/fnut.2026.1811125

**Published:** 2026-05-18

**Authors:** Mingyue Gao, Tingting Dong, Meirui Yuan, Hengheng Zhang, Xiaodan Zhang, Qiao Chen, Chen Liu

**Affiliations:** 1 Department of Radiotherapy, Air Force Medical Center, The Fourth Military Medical University, Beijing, China; 2 Department of Nursing, Air Force Medical Center, The Fourth Military Medical University, Beijing, China

**Keywords:** cancer pain, nomogram, nutritional risk, radiotherapy, severe lymphocytopenia

## Abstract

**Background:**

Radiotherapy-induced severe lymphocytopenia (SL) represents a critical immune-related toxicity associated with poor oncologic outcomes. While treatment-related factors have been extensively studied, the contribution of baseline nutritional risk and cancer-related pain to immune vulnerability during radiotherapy remains insufficiently explored. This study aimed to evaluate the role of nutrition- and symptom-related factors in SL and develop a clinically applicable predictive model.

**Methods:**

A retrospective cohort of 97 cancer patients receiving radiotherapy in 2022 was analyzed. Nutritional risk was assessed using the Nutritional Risk Screening 2002 (NRS-2002) before radiotherapy and at mid-treatment; the pretreatment result was used as the baseline nutritional risk variable in the predictive analyses, whereas the mid-treatment assessment was used for nutritional monitoring. Cancer pain was evaluated using the Numeric Rating Scale. Logistic regression identified independent predictors of SL (lymphocyte count < 0.5 × 10^9^/L). A nutrition-integrated nomogram was developed and internally validated using ROC analysis, calibration curves, and decision curve analysis (DCA).

**Results:**

Lymphocyte counts declined progressively during radiotherapy (*p* < 0.001), with 42.3% of patients developing SL. Multivariable analysis identified nutritional risk (OR = 3.53, *p* = 0.016), cancer pain (OR = 3.78, *p* = 0.037), and radiotherapy fractions (OR = 1.11 per fraction, *p* = 0.006) as independent predictors. Stratified analysis suggested a stronger association between cancer pain and SL among nutritionally at-risk patients. The nomogram demonstrated good discrimination (AUC = 0.77; C-index = 0.77), adequate calibration (Hosmer–Lemeshow *p* = 0.45), and meaningful clinical net benefit across a threshold range of 0.18–0.90.

**Conclusion:**

Nutritional risk and cancer pain independently contribute to radiotherapy-induced immune suppression. The proposed nutrition-integrated nomogram enables personalized immune risk assessment and supports early nutritional and symptom-targeted interventions to preserve immune competence during radiotherapy.

## Introduction

Malignant tumors remain one of the leading causes of death worldwide, accounting for millions of fatalities each year and placing a substantial burden on families and healthcare systems (1–5). With continued population aging and escalating environmental risk factors, cancer incidence is steadily increasing. Although conventional treatments such as surgery, radiotherapy, and chemotherapy have significantly prolonged patient survival, they still face considerable limitations in efficacy, tolerability, and the management of complications (6, 7). Among these modalities, radiotherapy plays a pivotal role in local tumor control and curative treatment across various solid malignancies. However, its immunosuppressive effects—particularly the detrimental impact on peripheral immune cells—have raised growing concern in clinical practice.

Radiotherapy-induced immunosuppression is primarily characterized by a marked reduction in peripheral blood lymphocytes, a condition referred to as radiation-induced lymphopenia (8). Lymphocytes play a vital role in immune surveillance, the elimination of abnormal cells, and the modulation of antitumor immune responses (9). Radiotherapy can directly damage lymphocyte DNA through radiation exposure, resulting in cell cycle arrest, inhibition of proliferation, and induction of apoptosis. In addition, secondary inflammatory responses triggered by radiotherapy stimulate the release of various cytokines, further disrupting lymphocyte production and function (10–14). Consequently, radiotherapy may compromise local tumor control, increase susceptibility to infections, delay postoperative recovery, and attenuate the efficacy of combination therapies such as immunotherapy and targeted therapy (15–17). Among these, severe lymphocytopenia (SL) is particularly recognized as a significant predictor of poor patient prognosis and has become one of the critical treatment-related toxicities requiring close monitoring in precision oncology.

Although previous studies have explored risk factors for SL from perspectives such as radiotherapy dose, irradiation site, and treatment duration, most have focused exclusively on treatment-related parameters, lacking a systematic evaluation of patients’ baseline characteristics (18). In clinical practice, increasing attention has been given to non-treatment-related variables—such as malnutrition and cancer pain—for their potential impact on immune function (19–22). In clinical practice, increasing attention has been given to non-treatment-related variables—such as malnutrition and cancer pain—for their potential impact on immune function. Poor nutritional status may impair hematopoietic capacity and lower immune response thresholds, thereby exacerbating lymphocyte production deficits (23). Chronic cancer pain, on the other hand, may activate neuro-immune pathways, leading to sustained inflammation and immunosuppression (24). These factors, often subtle and under-recognized, can compromise both radiotherapy tolerance and immune integrity, yet have been largely overlooked in SL-related research. Therefore, there is a pressing need for more comprehensive, multifactorial investigations to elucidate the role of non-treatment variables in radiotherapy-associated SL. Such efforts are essential for developing quantifiable and visualizable risk assessment tools to support personalized clinical decision-making.

In recent years, advances in precision medicine have promoted the widespread use of risk prediction models in oncology (25). Integrating multiple clinical variables into visualized predictive tools enables precise identification of high-risk patients and supports early interventions (18). In the context of SL, empirical assessment alone is no longer adequate. Developing a robust and interpretable model that integrates nutritional risk, cancer pain, and radiotherapy parameters is essential to optimize treatment decisions, reduce immunologic injury, and improve outcomes.

Against this background, this study investigates the mechanisms of SL during radiotherapy and identifies key predictive factors by integrating patient characteristics with treatment parameters. Nutritional risk and cancer pain, two clinically relevant but often overlooked variables, are incorporated into a multifactorial predictive model to improve individualized immune risk assessment. This model provides clinicians with a practical tool for early identification of high-risk patients and supports targeted interventions to preserve immune function and enhance treatment safety and quality of life.

## Materials and methods

### Study population

A total of 97 patients with malignant tumors who underwent radiotherapy at the Air Force Medical Center between January 2022 and December 2022 were enrolled in this study (Supplementary Figure S1). All enrolled patients met the following inclusion criteria: (1) histopathologically confirmed malignancy and eligibility for radiotherapy according to relevant clinical guidelines and expert consensus; (2) an Eastern Cooperative Oncology Group (ECOG) performance status score of < 2 and an expected survival time of > 6 months; and (3) full understanding of the study protocol and provision of written informed consent.

During the study period, 120 patients were initially screened. To minimize treatment-related heterogeneity, patients with unplanned radiotherapy interruption >1 week were excluded, as prolonged overall treatment time can alter delivered radiation intensity and has been associated with inferior oncologic outcomes (26–28). This cutoff was used to minimize treatment-related heterogeneity. In total, 23 patients were excluded: 8 for radiotherapy interruption >1 week, 9 for severe infection or significant hepatic and/or renal dysfunction, and 6 for psychiatric disorders or communication difficulties. The final analysis included 97 patients with complete clinical and follow-up data. The study was approved by the Ethics Committee of the Air Force Medical Center [AFMCSC (New Technology 2024-021-PJ01)].

### Radiotherapy protocol

All radiotherapy protocols were developed according to each patient’s specific tumor type, with reference to the *NCCN Clinical Practice Guidelines* (NCCN) and the *Chinese Society of Clinical Oncology* (CSCO) Guidelines for Common Malignant Tumors. Before radiotherapy, standardized immobilization was performed using head–neck-shoulder thermoplastic masks or body molds to ensure stable and reproducible positioning. Simulation scans were acquired with a Siemens Emotion 16-slice spiral CT simulator at a 3-mm slice thickness, covering both the lesion and potential regions at risk. The images were imported into the treatment planning system (TPS) for target delineation and dose design. Radiotherapy was delivered using either the Accuray TomoTherapy Hi·Art or the Elekta Synergy linear accelerator with 6-MV X-ray beams. For different tumor sites (e.g., head and neck, thoracic, and abdominopelvic malignancies), treatment indications, dose-fractionation regimens, and organ-at-risk (OAR) constraints strictly followed the corresponding disease-specific guidelines. Each treatment plan was finalized through multidisciplinary team (MDT) discussion among radiation oncologists and relevant specialists to achieve precise and individualized therapy.

### Nutritional assessment and intervention

Nutritional risk was assessed using the Nutritional Risk Screening 2002 (NRS-2002) by trained clinical dietitians or primary nurses at two prespecified time points: within 1 week before radiotherapy initiation and at mid-treatment, defined as approximately half of the planned radiotherapy course (29, 30). An NRS-2002 score ≥3 was considered nutritional risk and used as the threshold for nutritional intervention (29, 30). The pretreatment NRS-2002 result was used as the baseline nutritional risk variable in logistic regression, stratified analysis, and nomogram development, whereas the mid-treatment assessment was used for longitudinal nutritional monitoring and adjustment of supportive care during radiotherapy (31, 32). Patients with NRS-2002 ≥ 3 at either time point received nutritional intervention, including individualized dietary counseling, oral nutritional supplements, and symptom-oriented management for impaired intake, such as nausea or dysphagia. Treatment-related adverse effects and nutritional status were monitored weekly throughout radiotherapy (32).

### Data collection

During radiotherapy, the following data were collected for each patient: 1. Demographic information, including age, sex, marital status, educational level, and comorbidities (e.g., hypertension, diabetes mellitus, coronary artery disease). Cancer pain was assessed using the Numeric Rating Scale (NRS), with a score of ≥ 4 indicating moderate-to-severe pain (33). The use of opioid analgesics was also recorded. 2. Clinical characteristics, including body mass index (BMI), body weight before and after radiotherapy, primary tumor site, tumor stage, radiotherapy dose, number of fractions, concurrent therapy status, and systemic treatments administered during radiotherapy, including concurrent or sequential chemotherapy, immunotherapy (PD-1/PD-L1 inhibitors), and targeted therapy. Laboratory parameters, including serum albumin, prealbumin, hemoglobin, platelet count, neutrophil count, lymphocyte count, and monocyte count. Severe lymphocytopenia (SL) was defined as a lymphocyte count <0.5 × 10^9^/L during or after radiotherapy.

### Sample size estimation

Sample size adequacy for logistic regression was assessed using the pmsampsize package in R. The number of predictor variables was set to three, consistent with the final model, with an event rate of 42.3%, a C-index of 0.70, an *R*^2^ of 0.10, a shrinkage factor of 0.90, and a significance level (*α*) of 0.05. The minimum required sample size was estimated to be 119 patients, including at least 50 events. In the present study, 97 patients were actually included, among whom 41 experienced SL events, yielding an events-per-variable (EPV) value of approximately 13.7. This met the commonly accepted minimum criterion of EPV ≥ 10. However, the total sample size and number of events were still below the recommended requirements estimated using pmsampsize. Although the current sample size did not fully meet the minimum sample size requirement under the prespecified parameters, the number of events was considered sufficient to support preliminary exploratory modeling analysis. The relatively limited sample size may reduce statistical power, weaken the ability to detect weak-to-moderate associations, and affect the stability of model estimates, as reflected by wider confidence intervals for regression coefficients. Although the model showed good performance after internal validation using the bootstrap method, the potential risk of overfitting should still be noted. In summary, the model demonstrated good discrimination and calibration in the existing dataset; however, the results should be interpreted with caution and require further validation in larger, multicenter studies to assess their robustness and generalizability.

### Statistical analysis

All statistical analyses were performed using SPSS version 22.0 (IBM, United States). The normality of continuous variables was assessed using the Shapiro–Wilk test. Normally distributed data were expressed as mean ± SD and compared using the independent-sample *t*-test, whereas non-normally distributed data were expressed as median (interquartile range) and compared using the Mann–Whitney *U* test. Categorical variables were presented as counts (percentages) and compared using the chi-square test or Fisher’s exact test.

Stratified logistic regression and interaction analyses were performed to investigate potential interactions among key variables. Nutritional risk was selected *a priori* as the stratification variable (NRS-2002 score <3 vs. ≥3) because of its high prevalence and modifiability in patients undergoing radiotherapy. Stratified analyses were used to evaluate the associations of cancer pain and radiotherapy fractions with SL across nutritional strata. To further assess effect modification, an interaction term (“nutritional risk × cancer pain”) was introduced into the multivariate logistic regression model, and a significant coefficient (*p* < 0.05) was considered indicative of interaction. The biological rationale was that both malnutrition and chronic pain may activate the hypothalamic–pituitary–adrenal axis and increase inflammatory cytokine release, thereby aggravating immunosuppression (34).

For variable selection, univariate logistic regression was first performed, and variables with *p* < 0.10 were entered into the multivariate model to avoid omission of potential predictors. Independent predictors were retained at *p* < 0.05.

Violin plots were generated using ggplot2 (v3.3.6), and the nomogram was constructed and visualized using rms (v6.4.0). Model performance was evaluated using (1) the likelihood ratio test for overall significance, (2) the concordance index (C-index) for discrimination, (3) the area under the receiver operating characteristic curve (AUC) for predictive accuracy, with AUC values of 0.5–0.7, 0.7–0.9, and >0.9 indicating low, moderate, and high accuracy, respectively, and (4) the Hosmer–Lemeshow (H-L) test for calibration. Internal validation was performed using bootstrap resampling with 1,000 repetitions. For each bootstrap sample, the model was refitted and evaluated to obtain optimism-corrected estimates. Calibration was further assessed using the bootstrap-corrected calibration curve, and discrimination was assessed using the C-index and AUC (35, 36). Decision curve analysis was performed using rmda (v1.6) combined with ggplot2 to evaluate clinical net benefit across threshold probabilities.

## Results

### Dynamic changes in lymphocyte counts during radiotherapy

**Figure 1:**
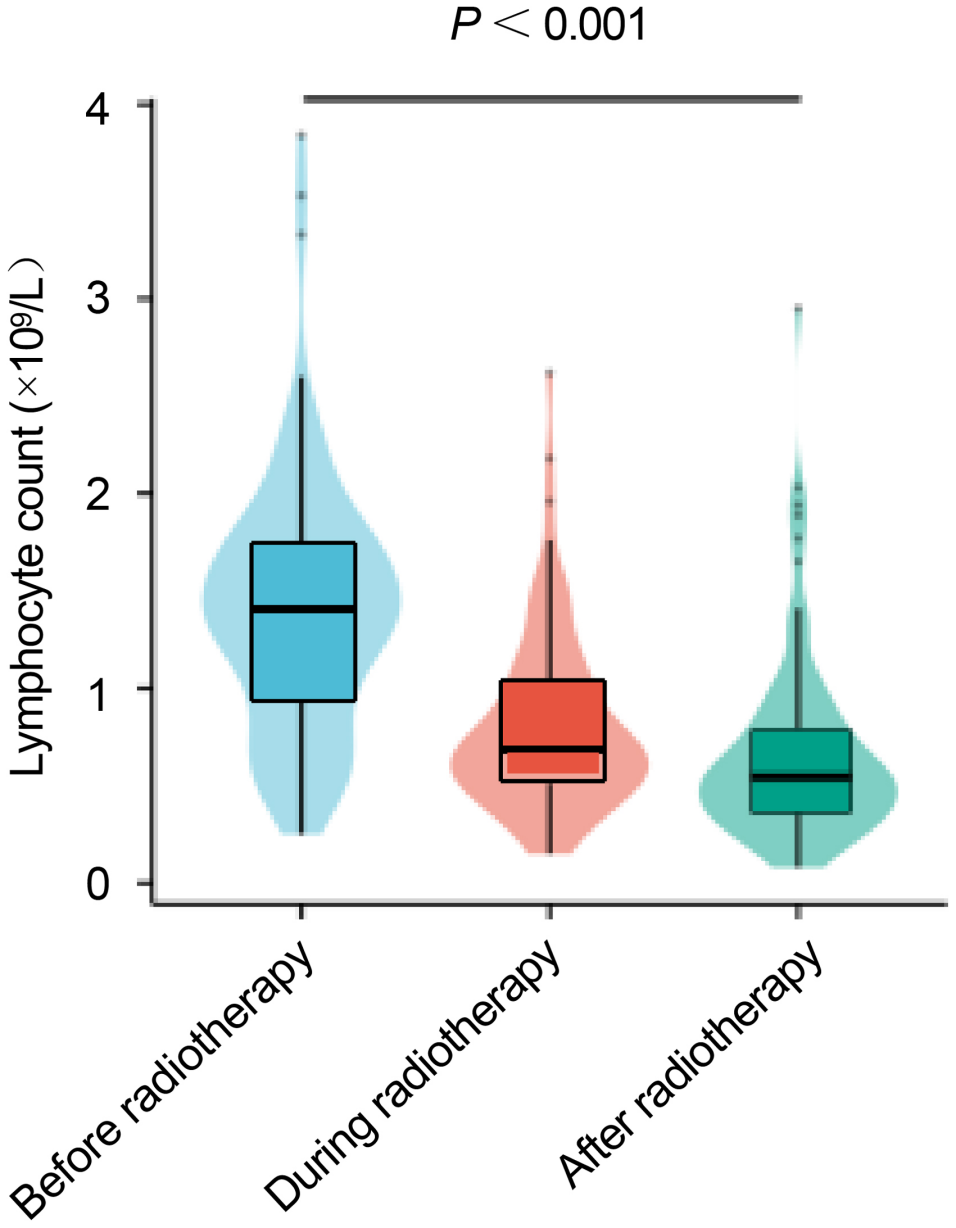
Violin plots of lymphocyte counts before, during, and after radiotherapy. Overall Friedman test, *p* < 0.001.

To evaluate the effect of radiotherapy on peripheral lymphocytes, lymphocyte counts at three time points (before, mid-, and post-radiotherapy) were compared among 97 patients. As the data were non-normally distributed and represented repeated measures, the nonparametric Friedman test was used for overall comparison. Lymphocyte counts showed a continuous decline during radiotherapy, with median values of 1.40 × 10^9^/L (0.93–1.73) before treatment, 0.68 × 10^9^/L (0.51–1.03) at mid-treatment, and 0.55 × 10^9^/L (0.36–0.79) after treatment. *Post hoc* pairwise comparisons with Bonferroni correction indicated significant differences between all time points (all *p* < 0.001; Figure 1). Peripheral lymphocyte counts decline markedly during radiotherapy.

### Comparative analysis of baseline characteristics in radiotherapy patients with SL

Before the first radiotherapy fraction. A total of 97 patients who underwent radiotherapy were included, of whom 41 (42.3%) developed SL and 56 (57.7%) did not. The baseline characteristics of the two groups are summarized in Table 1, and detailed comparisons of educational level are provided in Supplementary Table S1. Among the analyzed variables, tumor location (*p* = 0.023) and baseline nutritional risk (*p* = 0.007) differed significantly between groups. In the SL group, thoracic, abdominopelvic, and head and neck tumors accounted for 39.0, 34.1, and 24.4%, respectively, compared with 30.4, 33.9, and 12.5% in the non-SL group. Baseline nutritional risk was more frequent in the SL group than in the non-SL group (56.1% vs. 26.8%). No significant between-group differences were observed for age, sex, educational level, comorbidities, distant metastasis, BMI, or systemic therapy (all *p* > 0.05).

**Table 1:** Baseline characteristics of patients with and without severe lymphocytopenia after radiotherapy.

Characteristic	Non-severe lymphocytopenia (*n* = 56)	Severe lymphocytopenia (*n* = 41)	*p*-value
Age, median (IQR)	59 (51, 67.5)	61 (51, 64)	0.669
Gender, *n* (%)			0.405
Male	28 (50%)	17 (41.5%)	
Female	28 (50%)	24 (58.5%)	
Tumor location, *n* (%)			0.023
Head and neck	7 (12.5%)	10 (24.4%)	
Thoracic	17 (30.4%)	16 (39.0%)	
Abdominopelvic	19 (33.9%)	14 (34.1%)	
Others	13 (23.2%)	1 (2.4%)	
Comorbidities, *n* (%)			0.347
No	26 (46.4%)	23 (56.1%)	
Yes	30 (53.6%)	18 (43.9%)	
Distant metastasis, *n* (%)			0.225
No	30 (53.6%)	27 (65.9%)	
Yes	26 (46.4%)	14 (34.1%)	
Baseline nutritional risk, *n* (%)			0.007
No	41 (73.2%)	18 (43.9%)	
Yes	15 (26.8%)	23 (56.1%)	
BMI, mean ± SD	23.34 ± 3.64	23.10 ± 3.357	0.741
Systemic therapy (any), *n* (%)	22 (39.3%)	16 (39.0%)	0.970

Regarding systemic therapy, 33.0% (32/97) of the cohort received concurrent or sequential chemotherapy, predominantly platinum-based regimens; 11.3% (11/97) received immunotherapy, and 8.2% (8/97) received targeted therapy. The overall use of systemic therapy was comparable between the non-SL and SL groups, with rates of 39.3% (22/56) and 39.0% (16/41), respectively (*p* = 0.970).

### Analysis of influencing factors for SL in radiotherapy patients

To further investigate factors associated with SL, 12 candidate variables were included in the analysis: nutritional risk (NRS-2002), nutritional intervention, activities of daily living (ADL), cancer pain, concurrent chemotherapy, number of radiotherapy fractions, hemoglobin, albumin, prealbumin, neutrophil-to-lymphocyte ratio (NLR), platelet-to-lymphocyte ratio (PLR), and lymphocyte-to-monocyte ratio (LMR). Univariable logistic regression showed that nutritional risk (OR = 3.493, 95% CI: 1.486–8.209, *p* = 0.004), ADL (OR = 0.393, 95% CI: 0.139–1.109, *p* = 0.078), cancer pain (OR = 2.425, 95% CI: 0.919–6.397, *p* = 0.074), concurrent chemotherapy (OR = 2.185, 95% CI: 0.947–5.042, *p* = 0.067), number of radiotherapy fractions (OR = 1.092, 95% CI: 1.031–1.157, *p* = 0.003), and PLR (OR = 1.005, 95% CI: 1.001–1.010, *p* = 0.020) were potentially associated with SL (Table 2). Based on the predefined selection criteria, these variables, together with nutritional intervention, were entered into the multivariable logistic regression model. In multivariable analysis, nutritional risk (OR = 3.533, 95% CI: 1.292–10.36, *p* = 0.0164), cancer pain (OR = 3.775, 95% CI: 1.119–14.04, *p* = 0.0373), and number of radiotherapy fractions (OR = 1.106, 95% CI: 1.033–1.193, *p* = 0.0056) remained independent predictors of SL, whereas ADL, concurrent chemotherapy, nutritional intervention, and PLR were not significant in the multivariable model (Table 3).

**Table 2:** Univariable logistic regression analysis for severe lymphocytopenia after radiotherapy.

Variables	Univariate analysis	
	OR (95% CI)	*p*-value
Nutritional risk (NRS-2002)	3.493 (1.486–8.209)	0.004
Received nutritional intervention	1.908 (0.785–4.638)	0.154
ADL	0.393 (0.139–1.109)	0.078
Cancer pain	2.425 (0.919–6.397)	0.074
Concurrent chemotherapy	2.185 (0.947–5.042)	0.067
Number of radiotherapy sessions	1.092 (1.031–1.157)	0.003
Hemoglobin	0.997 (0.977–1.017)	0.758
Albumin	1.028 (0.917–1.151)	0.637
Prealbumin	1.000 (0.994–1.007)	0.918
NLR	1.057 (0.968–1.154)	0.218
PLR	1.005 (1.001–1.010)	0.020
LMR	0.919 (0.758–1.114)	0.388

ADL, activities of daily living; NLR, neutrophil-to-lymphocyte ratio; PLR, platelet to lymphocyte ratio; LMR, lymphocyte-to-monocyte ratio.

**Table 3:** Multivariable logistic regression analysis for severe lymphocytopenia after radiotherapy.

Variables	Multivariate analysis	
	OR (95% CI)	*p*-value
Nutritional risk (NRS-2002)	3.533 (1.292–10.36)	0.0164
Received nutritional intervention	0.9703 (0.3108–2.977)	0.9579
ADL	0.3969 (0.09583–1.403)	0.1701
Cancer pain	3.775 (1.119–14.04)	0.0373
Concurrent chemotherapy	1.863 (0.6595–5.393)	0.2415
Number of radiotherapy sessions	1.106 (1.033–1.193)	0.0056
PLR	1.004 (1.000–1.009)	0.1021

ADL, activities of daily living; PLR, platelet to lymphocyte ratio.

### Stratified regression analysis of radiotherapy patients with SL

**Figure 2:**
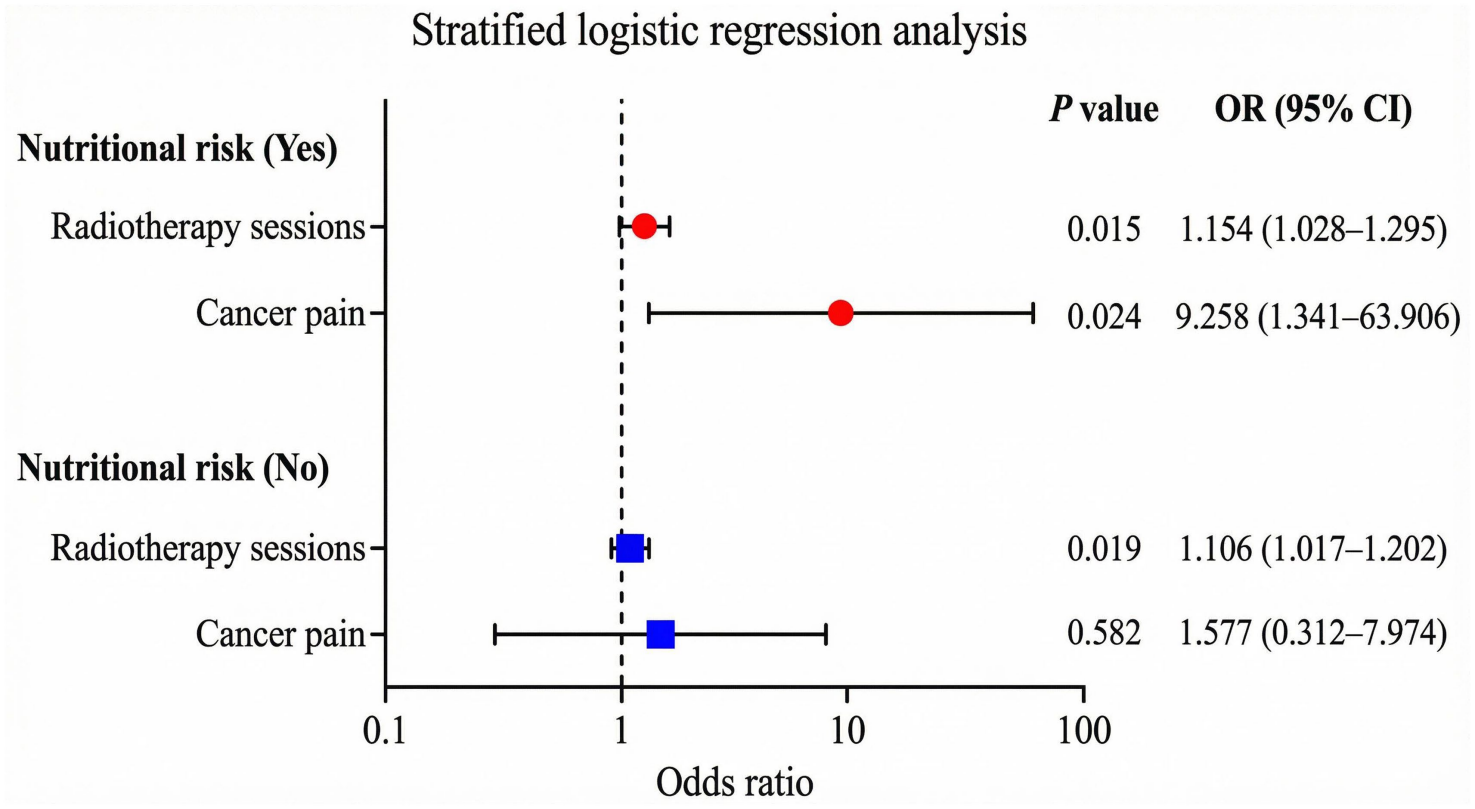
Stratified logistic regression analysis of risk factors for SL.

To explore potential interactions between predictors, we conducted stratified regression and interaction analyses. In the stratified analysis based on nutritional risk status, the number of radiotherapy fractions remained a significant risk factor for SL in both subgroups. Specifically, among patients with nutritional risk (NRS 2002 score ≥ 3), the OR for radiotherapy fractions was 1.154 (95% CI: 1.028–1.295, *p* = 0.015), while in the well-nourished group (NRS < 3), the OR was 1.106 (95% CI: 1.017–1.202, *p* = 0.019), both statistically significant. However, the interaction test between radiotherapy fractions and nutritional risk was not significant (OR = 1.012, 95% CI: 0.889–1.151, *p* = 0.859), indicating that the effect of radiotherapy fractions on SL was consistent regardless of nutritional status. In contrast, cancer pain was identified as an independent predictor of SL only in the nutritional risk subgroup (OR = 9.258, 95% CI: 1.341–63.906, *p* = 0.024), but not in the well-nourished group (OR = 1.577, 95% CI: 0.312–7.974, *p* = 0.582; Table 4; Figure 2). The interaction model showed a non-significant yet suggestive trend between cancer pain and nutritional risk (interaction OR = 5.147, 95% CI: 0.539–49.179, *p* = 0.155), supporting a hypothesis that cancer pain may exert a stronger effect on SL in patients with poor nutritional status. This trend should be interpreted with caution and verified in larger studies. Additionally, there was no significant interaction between cancer pain and radiotherapy fractions (interaction OR = 1.077, 95% CI: 0.909–1.276, *p* = 0.394), indicating that the effect of radiotherapy frequency on lymphopenia is independent of cancer pain status.

**Table 4:** Stratified logistic regression analysis for severe lymphocytopenia after radiotherapy.

Variables	Multivariate analysis	
	OR (95% CI)	*p*-value
Nutritional risk present (NRS-2002 ≥ 3)		
Radiotherapy sessions	1.154 (1.028–1.295)	0.015
Cancer pain	9.258(1.341–63.906)	0.024
No nutritional risk (NRS-2002 < 3)		
Radiotherapy sessions	1.106 (1.017–1.202)	0.019
Cancer pain	1.577 (0.312–7.974)	0.582
Interaction terms		
Radiotherapy sessions × Nutritional risk	1.012 (0.889–1.151)	0.859
Cancer pain × Nutritional risk	5.147 (0.539–49.179)	0.155
Cancer pain × Radiotherapy sessions	1.077 (0.909–1.276)	0.394

### Risk prediction and evaluation of SL in radiotherapy patients

**Figure 3:**
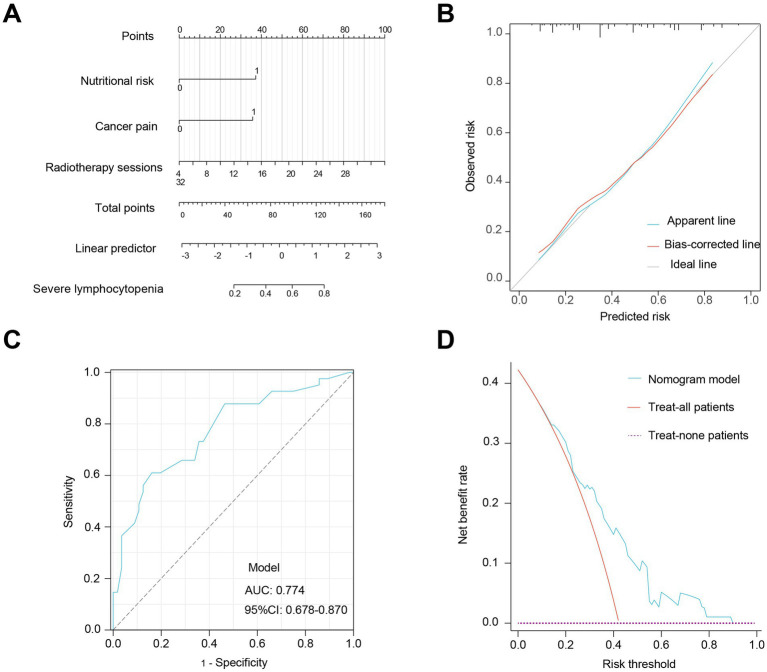
Risk prediction and evaluation of SL. **(A)** Nomogram model for quantifying the risk of SL. **(B)** Calibration curve of the model. **(C)** ROC curve analysis. **(D)** DCA of the nomogram model.

A nomogram model was constructed based on multivariable logistic regression to quantitatively predict the risk of SL. Among the three predictors included in the model, the number of radiotherapy fractions contributed the largest point range, indicating it as the most influential factor—an increased number of sessions corresponded to a higher risk of SL. Nutritional risk and cancer pain contributed 38 and 36 points, respectively. Combined, these factors enabled individualized risk estimation: the higher the total nomogram score, the greater the predicted probability of SL. For example, a total score exceeding 128 points corresponded to an estimated SL risk of up to 80% (Figure 3A).

To evaluate the stability and performance of the predictive model, variance inflation factor (VIF) analysis was conducted for the three included variables (nutritional risk, cancer pain, and radiotherapy fractions). The VIF values were 1.05, 1.12, and 1.16, all of which are well below the commonly accepted threshold of 10, indicating no multicollinearity. The likelihood ratio test demonstrated that the overall model was statistically significant (*χ*^2^ = 24.43, *p* < 0.001). Model discrimination was assessed using the C-index, which was 0.77, indicating good discriminative ability. The calibration plot showed close agreement between the apparent, bias-corrected, and ideal lines, indicating acceptable internal stability of the nomogram after bootstrap validation. The Hosmer-Lemeshow test also supported good calibration (*χ*^2^ = 7.84, *p* = 0.45; Figure 3B). ROC analysis yielded an AUC of 0.77 (95% CI: 0.68–0.87), further supporting the model’s predictive performance (Figure 3C). DCA demonstrated clear clinical benefit when the risk threshold ranged from 0.18 to 0.90, indicating strong practical utility of the nomogram model in real-world settings (Figure 3D).

## Discussion

This study analyzed dynamic changes in peripheral blood lymphocyte counts in 97 cancer patients undergoing radiotherapy and showed a sustained decline throughout treatment, with a 60.7% reduction at the end of radiotherapy relative to baseline. This finding is consistent with previous reports of radiation-induced lymphocyte apoptosis (12, 37) and further supports the strong immunosuppressive effect of radiotherapy. Against this background, three independent predictors of SL were identified, namely nutritional risk (NRS-2002 score ≥3), cancer pain, and the number of radiotherapy fractions. A nomogram incorporating these variables achieved a C-index and AUC of 0.77, indicating good discriminative ability. By integrating nutritional and symptom-related factors with treatment exposure, this model extends risk assessment beyond conventional treatment parameters and supports a more patient-centered approach to predicting radiotherapy-related immunotoxicity.

In contrast to previous studies that primarily focused on radiotherapy dose, irradiation field, or fractionation schemes, this study highlights the combined contribution of treatment exposure and baseline patient status to immune vulnerability during radiotherapy. Each additional radiotherapy fraction was associated with an 11% increase in the risk of SL, consistent with prior reports. Ng et al. reported an average decrease in absolute lymphocyte count of approximately 0.05 × 10^9^/L per radiotherapy session in patients with head and neck cancer (11). D’Auria et al. observed greater preservation of peripheral lymphocytes with hypofractionated regimens than with more prolonged conventional schedules (38), and Zhao et al. further suggested that shortening the radiotherapy course may facilitate lymphocyte recovery (39). In our cohort, the median number of radiotherapy fractions was 20 and the median total dose was 40 Gy. The effect of radiotherapy fractions, however, should not be interpreted in isolation. Radiation-induced lymphopenia is also influenced by dose-related factors, including low-dose exposure of the heart and lungs, mean organ dose, and blood-dose surrogates that estimate irradiation of circulating immune cells (40–42). The association between fraction number and SL may therefore partly reflect unmeasured differences in total dose, dose per fraction, target volume, and dose distribution, rather than a purely numerical fraction effect. This interpretation is consistent with the sustained decline in lymphocyte counts observed across the radiotherapy course in the present study.

Among host-related predictors, BMI was not significantly associated with SL, whereas baseline nutritional risk defined by NRS-2002 remained independently associated with its occurrence. This difference may reflect the fact that NRS-2002 captures nutritional compromise and disease burden more comprehensively than BMI alone (41–43). The relationship between nutritional status and radiation-related outcomes is not entirely consistent across the current literature. Although malnutrition during radiotherapy is generally associated with poorer treatment tolerance and greater supportive-care needs (31), its association with specific treatment-related endpoints varies across studies. Recent evidence suggests that nutritional interventions can improve some nutritional and toxicity outcomes in patients receiving radiotherapy or chemoradiotherapy, but these effects are not uniform across all tumor types or endpoints (44, 45). Our findings therefore support standardized baseline nutritional screening before radiotherapy, while indicating that the contribution of nutritional status to radiation-related immune toxicity should be interpreted in a disease- and endpoint-specific framework. Cancer pain also remained an independent predictor of SL, and its association appeared stronger in nutritionally at-risk patients, although the interaction term did not reach statistical significance. This pattern should be regarded as hypothesis-generating and requires validation in larger, more homogeneous cohorts.

Another notable aspect of this study was the inclusion of cancer pain in the predictive model for SL and the exploration of its potential interaction with nutritional risk. Stratified analysis suggested that the association between cancer pain and SL was stronger among patients with nutritional risk, although the interaction term did not reach statistical significance. This finding should therefore be interpreted as hypothesis-generating rather than confirmatory. Chronic pain is increasingly recognized as a neuroimmune condition characterized by reciprocal signaling between immune cells and the nervous system (46). In the cancer setting, pain-related immune dysregulation may represent an additional source of systemic vulnerability, potentially interacting with other host-related factors such as malnutrition (47). Consistent with this possibility, post-radiotherapy lymphocyte counts were lower in patients with cancer pain in our cohort. These observations support further investigation of the nutrition–pain–immunity axis in larger and more homogeneous radiotherapy cohorts.

The nomogram developed in this study provides a practical tool for individualized risk estimation of severe lymphocytopenia during radiotherapy. By integrating baseline nutritional risk, cancer pain, and radiotherapy fractions, the model extends risk assessment beyond conventional treatment parameters. Decision curve analysis suggested potential clinical utility across threshold probabilities above 0.18. A major strength of the model is the inclusion of clinically accessible nutrition-related and symptom-related variables. In practice, the model may help identify patients who warrant closer supportive care during radiotherapy, including nutritional assessment, pain management, and more intensive immune monitoring. More broadly, this risk-stratified approach may support more targeted delivery of supportive interventions in routine radiotherapy care.

Despite the scientific value of this study, several limitations should be acknowledged. First, this was a single-center retrospective study with a relatively small sample size, which may introduce selection bias. In addition, the lack of long-term follow-up data precludes the evaluation of the impact of severe lymphopenia on survival outcomes and tumor recurrence. Second, the study cohort included patients with various tumor types and treatment intents, resulting in substantial heterogeneity in disease stage, target volumes, and irradiation fields. Such heterogeneity may lead to differences in radiation-related immune injury and thereby affect the stability of the model. Third, although nutritional status, cancer pain, and radiotherapy fractions were included, systemic therapy regimens and key dosimetric parameters could not be comprehensively controlled in this multi-tumor retrospective cohort. Radiotherapy fractions should therefore not be interpreted independently of dose-related factors. Recent evidence suggests that radiation-induced lymphopenia is influenced not only by treatment duration, but also by low-dose exposure of thoracic organs, mean organ dose, and blood-dose surrogates that estimate irradiation of circulating immune cells. The association between radiotherapy fractions and severe lymphopenia observed in this study may thus partly reflect unmeasured differences in total dose, dose per fraction, target volume, and dose distribution, rather than a purely numerical fraction effect. Residual confounding is therefore unavoidable, and severe lymphopenia cannot be attributed to any single factor in the present dataset (40, 48–50). Fourth, immune-related biological indicators such as lymphocyte subsets and immune checkpoint molecule expression were not collected, and pain and nutritional assessments partially relied on subjective scales, introducing potential measurement bias. Fifth, the predictive model has not undergone external validation. Given the relatively small sample size and the heterogeneity of the cohort, its generalizability and clinical applicability remain limited. Further validation in large-scale, homogeneous, multi-center cohorts with comprehensive treatment-related data is required.

Building upon these findings, future research may advance in several directions. First, multicenter prospective studies with larger sample sizes are needed to validate the stability and external applicability of the model. Second, priority should be given to external validation in homogeneous cohorts with a single tumor type, clearly defined treatment intent, and standardized radiotherapy techniques and dose prescriptions, while systematically incorporating dose–volume metrics (e.g., ILV, V5, V10, MLD) to enhance the model’s internal validity and causal interpretability. Third, integrating multidimensional immunological data, such as lymphocyte subset profiling, cytokine signatures, and immune checkpoint molecule expression, may provide deeper insights into the mechanisms of radiation-related immune suppression. Fourth, the inclusion of radiomics, metabolomics, and other multimodal omics features, coupled with machine-learning approaches, may further optimize variable selection and improve predictive performance. Fifth, interventional randomized controlled trials based on the proposed model are warranted, particularly to evaluate the actual clinical benefits of combined strategies such as nutritional support and pain management on lymphocyte recovery. Sixth, incorporating health economic evaluations may help determine the cost–effectiveness of applying this model in radiotherapy supportive care and aid its clinical translation. Notably, the second to fourth weeks of radiotherapy represent a critical window of rapid lymphocyte decline; therefore, future studies should explore optimal strategies for high-frequency dynamic immune monitoring and timely intervention during this period to better prevent or mitigate severe lymphopenia.

## Conclusion

**Figure 4:**
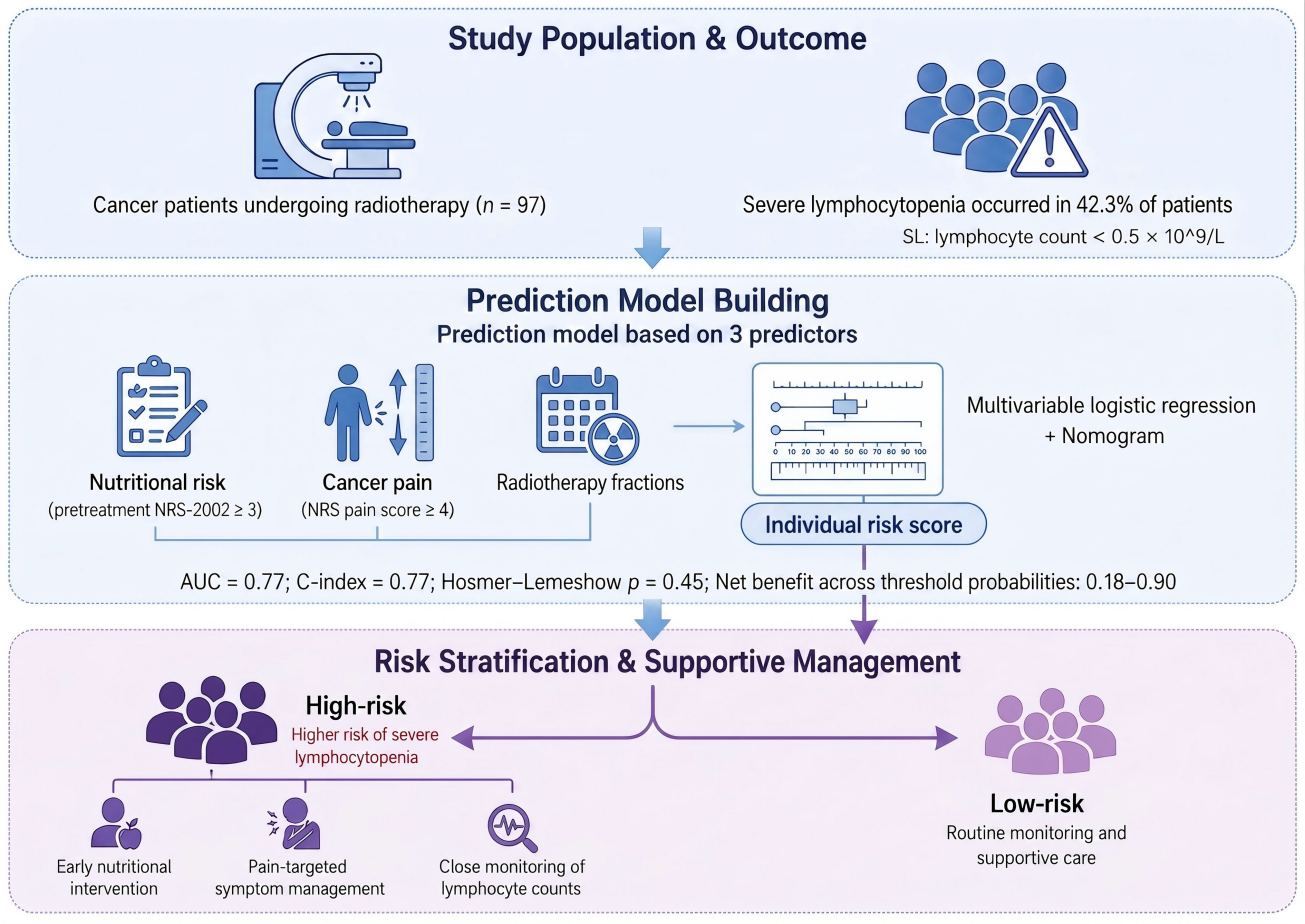
Development of an individualized model to predict the risk of radiotherapy-associated severe lymphocytopenia based on three clinical indicators.

This study investigated the incidence and determinants of severe lymphocytopenia (SL) during radiotherapy in a cohort of 97 patients with malignant tumors. A nomogram incorporating baseline nutritional risk (NRS-2002 score ≥3), radiotherapy fractions, and cancer pain showed moderate discrimination and acceptable calibration, with a C-index and AUC of 0.77. Decision curve analysis further suggested potential clinical utility across a threshold probability range of 0.18–0.90. These findings indicate that the model may help identify patients at increased risk of SL during radiotherapy. However, given the single-center design, limited sample size, and heterogeneity in tumor types and treatment regimens, further validation in larger and more homogeneous cohorts is required before broader clinical application (Figure 4).

## Data Availability

The original contributions presented in the study are included in the article/Supplementary material, further inquiries can be directed to the corresponding author/s.

## Supplementary material

Supplementary Figure S1 Flowchart of patient enrollment and exclusion criteria in the radiotherapy cohort study.

## Glossary

ADL: Activities of Daily Living
AUC: Area under the Curve
BMI: Body Mass Index
C-index: Concordance Index
CI: Confidence Interval
CSCO: Chinese Society of Clinical Oncology
DCA: Decision Curve Analysis
ECOG: Eastern Cooperative Oncology Group
EPV: Events-Per-Variable
H-L: Hosmer-Lemeshow
IL-6: Interleukin-6
IQR: Interquartile Range
LMR: Lymphocyte-to-Monocyte Ratio
MDT: Multidisciplinary Team
NCCN: National Comprehensive Cancer Network
NLR: Neutrophil-to-Lymphocyte Ratio
NRS: Numeric Rating Scale
NRS 2002: Nutritional Risk Screening 2002
ONS: Oral Nutritional Supplements
OR: Odds Ratio
PLR: Platelet-to-Lymphocyte Ratio
ROC: Receiver Operating Characteristic
SL: Severe Lymphocytopenia
TPS: Treatment Planning System
VIF: Variance Inflation Factor
