# Correction: Nutritional risk and cancer pain as determinants of radiotherapy-induced severe lymphocytopenia: development and validation of a nutrition-integrated predictive nomogram

**DOI:** 10.3389/fnut.2026.1884981

**Published:** 2026-07-15

**Authors:** Mingyue Gao, Tingting Dong, Meirui Yuan, Hengheng Zhang, Xiaodan Zhang, Qiao Chen, Chen Liu

**Affiliations:** 1 Department of Radiotherapy, Air Force Medical Center, The Fourth Military Medical University, Beijing, China; 2 Department of Nursing, Air Force Medical Center, The Fourth Military Medical University, Beijing, China

**Keywords:** cancer pain, nomogram, nutritional risk, radiotherapy, severe lymphocytopenia

There was a mistake in the Graphical Abstract as published. It should have been published as Figure 4. This has been removed from the article.

There was a mistake in Figure 4 as published. The previous version of Figure 4 was inadvertently retained in the published article instead of the updated version requested during the proofing stage.

The correct Figure 4 appears below.

**Figure 4:**
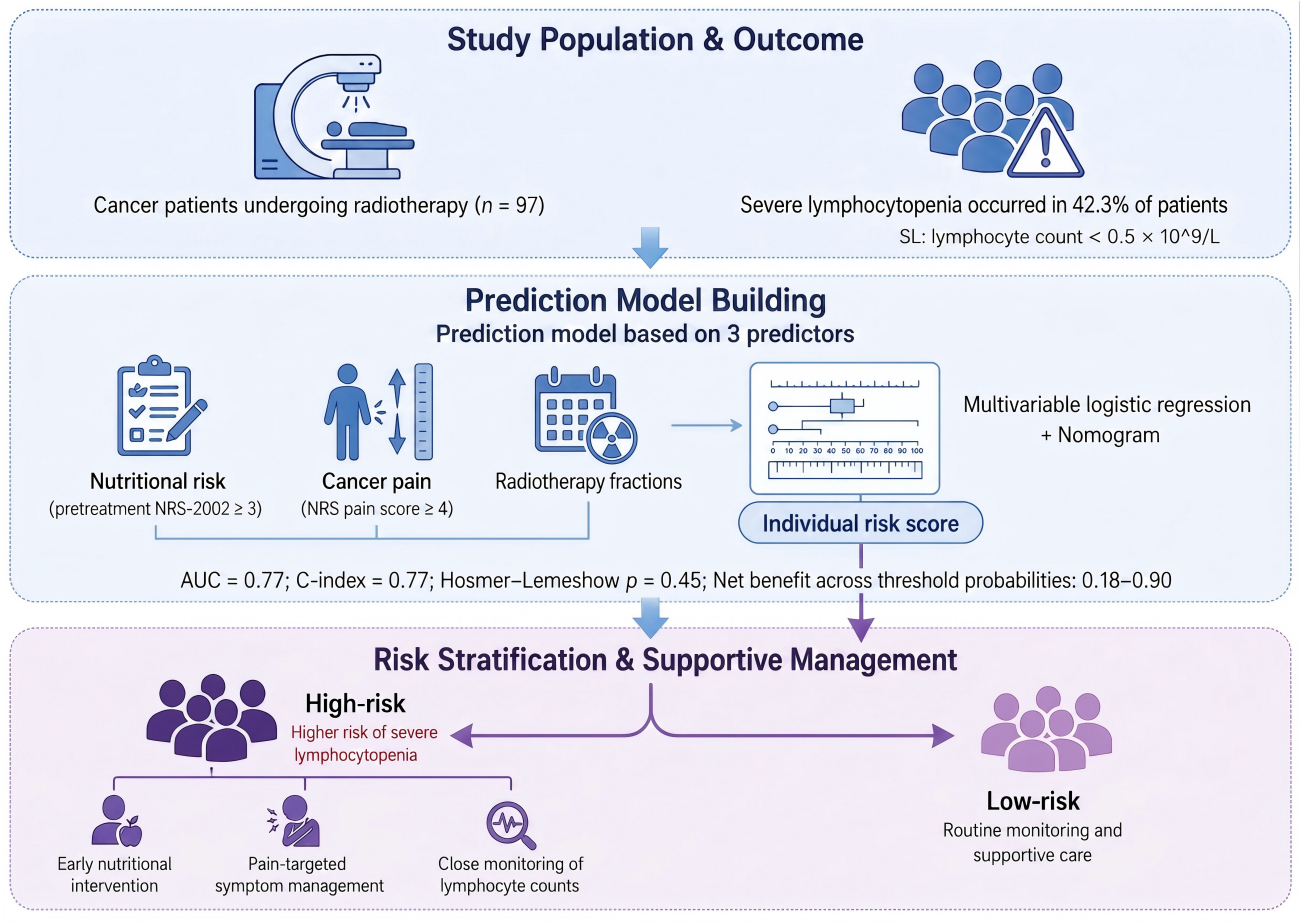
Development of an individualized model to predict the risk of radiotherapy-associated severe lymphocytopenia based on three clinical indicators. (1) the Individual Risk Score component; (2) model performance metrics, including AUC, C-index, Hosmer–Lemeshow *p*-value, and net benefit across threshold probabilities; (3) the Risk Stratification and Supportive Management section illustrating clinical application of the model.

The original version of this article has been updated.

