# Supplementary figures and images for: Nutritional risk and cancer pain as determinants of radiotherapy-induced severe lymphocytopenia: development and validation of a nutrition-integrated predictive nomogram
Source: Front Nutr. 2026 May 18;13:1811125. doi: 10.3389/fnut.2026.1811125 (PMC13223039; doi:10.3389/fnut.2026.1811125)

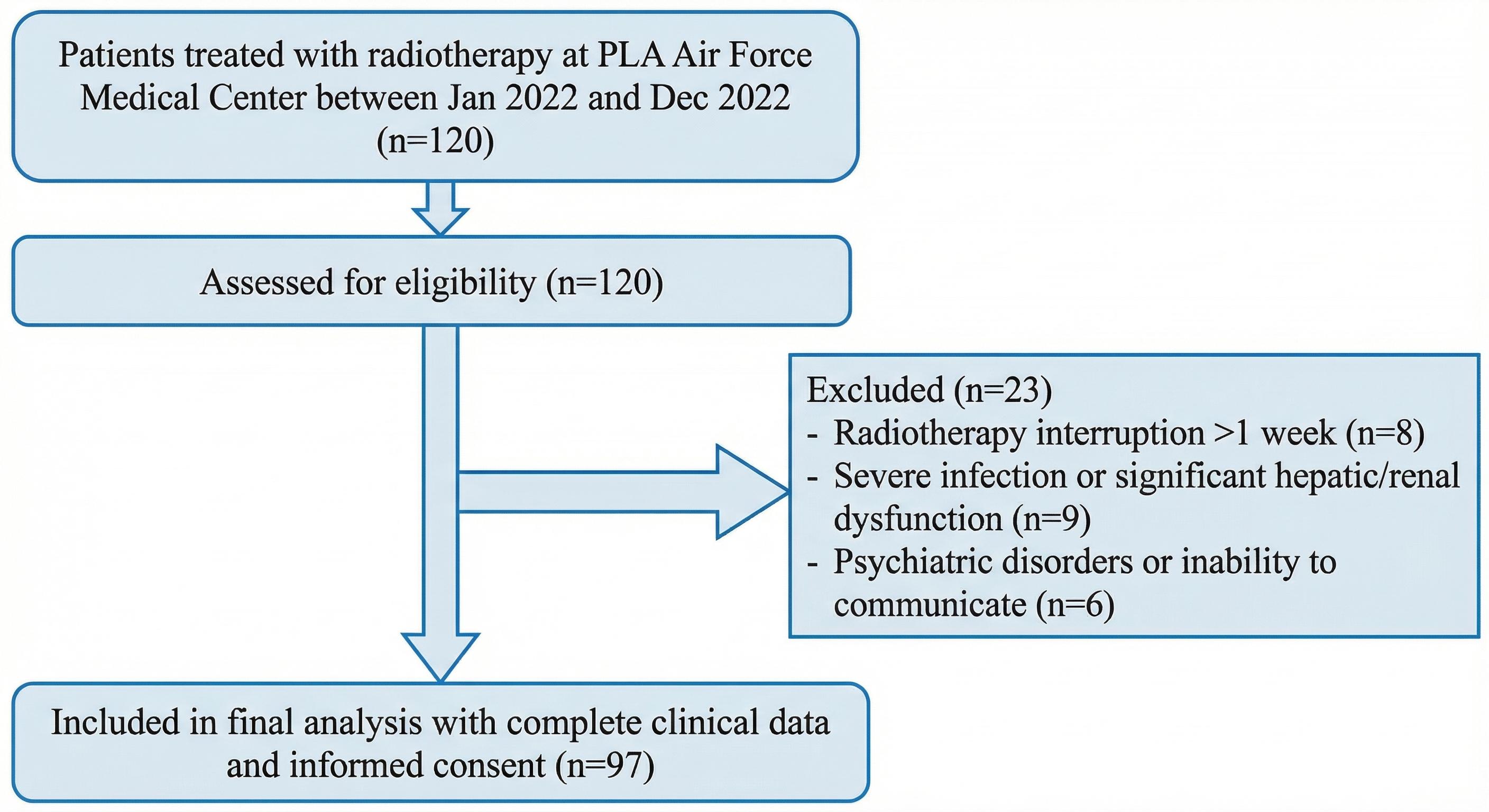

Supplement: Supplementary Figure S1 — Flowchart of patient enrollment and exclusion criteria in the radiotherapy cohort study. [file Image_1.JPEG]
