# Supplementary material for: Nutritional risk and cancer pain as determinants of radiotherapy-induced severe lymphocytopenia: development and validation of a nutrition-integrated predictive nomogram
Source: Front Nutr. 2026 May 18;13:1811125. doi: 10.3389/fnut.2026.1811125 (PMC13223039; doi:10.3389/fnut.2026.1811125)
Supplement: Supplementary file 2 [file Table_1.docx]

**Table S1. Comparison of educational level between patients with and without severe lymphocytopenia**

| **Characteristic** | **Non-Severe lymphocytopenia (n=56)** | **Severe lymphocytopenia (n=41)** | ***P*-value** |
| --- | --- | --- | --- |
| Education level, n (%) |  |  | 0.377 |
| Primary school or below | 7 (12.5%) | 7 (17.1%) |  |
| Junior high school | 16 (28.6%) | 15 (36.6%) |  |
| Senior high school / technical secondary school | 20 (35.7%) | 8 (19.5%) |  |
| College or above | 13 (23.2%) | 11 (26.8%) |  |

Note: Data are presented as n (%). P value was calculated using the chi-square test or Fisher’s exact test, as appropriate.
